# Self-rated health changes of individuals in same-sex compared to different-sex relationships: a longitudinal study

**DOI:** 10.1186/s12889-022-13283-w

**Published:** 2022-04-29

**Authors:** Yiwen Wang

**Affiliations:** Department of Sociology & Population Research Center, The University of Texas at Austin, 305 E 23rd St, A1700, RLP 2.214A, Austin, TX 78712-1086 USA

**Keywords:** Longitudinal data, Minority stress, United States, Same-sex relationships, Self-rated health

## Abstract

**Background:**

Prior scholarship on same-sex relationships and health has primarily relied on cross-sectional data, leaving a number of unanswered questions about health changes of same-sex couples over time. This study examined the self-rated health statuses and changes of individuals in same- and different-sex cohabitations and marriages over time (2014–2017).

**Methods:**

Data were drawn from the 2014 panel of the Survey of Income and Program Participation (SIPP), a nationally representative and longitudinal study (*N* = 23,297) in the United States. Mixed- and fixed-effects regression models were performed to investigate the self-rated health changes of individuals in same-sex compared to different-sex relationships.

**Results:**

Results show that same-sex married individuals report a faster decline in self-rated health compared to different-sex married counterparts despite similar initial health statuses. Similarly, same-sex cohabitors also exhibit a more rapid health decline as compared to different-sex cohabitors.

**Conclusions:**

The results point to health change disadvantages experienced by same-sex married and cohabiting individuals during the study period. The findings from this study advance scholarly knowledge on the health changes of individuals in marginalized intimate relationships and highlight the importance of studying sexual minorities’ health using longitudinal data.

## Background

Involvement in intimate relationships is a fundamental human need, regardless of sexual orientation [1]. However, while the pursuit of intimacy is equally meaningful and important for sexual minorities and heterosexuals, gay and lesbian individuals experience romantic relationships within a broader social context that devalues and diminishes their pursuit of intimacy and puts them at risk of poor health and well-being [1–3]. Despite a growing body of research evaluating health similarities and disparities between individuals in same-sex and different-sex relationships [4–7], the available evidence relies almost entirely on cross-sectional data to compare the health of same- and different-sex couples at a single point in time. Scholarly understanding of same-sex relationships and health changes in nationally representative studies is sorely lacking.

Two conceptual frameworks can be used to hypothesize about the health changes of individuals in same-sex and different-sex cohabitations and marriages—the minority stress proliferation framework and the gender-as-relational framework.

### The minority stress proliferation framework

Individuals in same-sex relationships may experience faster health decline based on minority stress and stress proliferation theories. The minority stress theory posits that sexual minorities experience social stressors (e.g. stigma and discrimination; expectation of rejection) related to their sexual identity [8]. In addition to individual-level minority stressors, partnered sexual minorities face unique couple-level stressors (e.g. internalized devaluation of one’s own relationship; concealment of same-sex relationship) associated with being in a socially devalued intimate relationship [9]. The experiences of homophobia, discrimination, and poor health care have been found to be negatively associated with physical and mental health as well as health behaviors among sexual minority individuals [10–12].

Despite a large body of health disparity literature documenting the long-term deleterious health impacts of being identified by a socially disadvantaged status, how sexual minority relationship status influences health over time remains an under-investigated question. The stress proliferation theory is particularly helpful when theorizing about sexual minority relationship status and health changes over time. The theory emphasizes that discriminatory experiences and stressors linked to a socially disadvantaged status may repeat and accrue across time to accelerate health decline among socially marginalized groups [13]. Empirical studies have found the cumulative disadvantages and racial discrimination Blacks experience over time lead them to age faster and die earlier than Whites [14, 15]. Immigration and health researchers also observed an unhealthy assimilation phenomenon. That is, although immigrants arrive at the destination country with better health than the native-born population, their initial health advantage declines with their stay [16]. Experiences of discrimination, accumulative stress, restricted access to quality health care, unhealthy stress coping behaviors, and poor socioeconomic conditions combine to erode immigrants’ health over time. Similarly, the individual- and couple-level minority stressors same-sex couples experience in daily life may accumulate over time and beget other stressors, leading to a more rapid health deterioration for them at the population level.

Previous research showing that same-sex cohabiting elders fare worse than different-sex married and even different-sex cohabitors provides some support for the possible effects of lifelong experiences of minority stress on same-sex couples’ health [4]. In a one-year follow up study, Frost and colleagues found that experiences of stressful events stemming from minority status were associated with worse physical health a year after the experience among sexual minority individuals [17]. Relatedly, recent studies also indicate that even if legal marriage is available to same-sex couples, it may still not be fully supported by the families, friends, colleagues, and employers of same-sex couples. The continuing backlash against sexual minority rights, especially during the Trump era (e.g., Masterpiece Cakeshop Ltd. v. Colorado Civil Rights Commission), may prevent same-sex couples from benefiting as much from marriage as different-sex couples in the United States. The stigma and discrimination against same-sex couples may discourage them from translating institutional resources and social support associated with marriage into health benefits [18]. Although the extant empirical research points to short- and long-term detrimental effects of minority stressors on health, no study has directly evaluated the longitudinal impact of same-sex relationships on health in population-based data.

### The gender-as-relational framework

Alternatively, a gender-as-relational framework suggests that men and women act differently in intimate relationships depending on whether they are interacting with a man or woman [19, 20]. The gender-as-relational framework advances the previously dichotomous view of men and women in relationships by recognizing gender as dynamic and relational. This approach allows researchers to consider if, and how, gender scripts operate differently across gendered relational contexts. For example, a woman married to a woman may adhere less strongly to traditional gender norms when compared with a woman married to a man [21]. Men and women in same-sex relationships are more likely to draw on gender similarities rather than differences, which may have implications for their relationship dynamics and health outcomes [20]. Indeed, empirical studies found that men and women in same-sex marriages communicate and negotiate more effectively [22], divide housework more equally [23], provide more caregiving when their spouse is ill [24, 25], and devote more effort to monitoring their partners’ health behaviors compared to men and women in different‐sex marriages [26]. Given that same-sex couples share similar relationship quality and stability as different-sex couples [27–29], and perhaps more egalitarian and productive relationship dynamics [30, 31], they may show similar or even better health changes when compared to different-sex counterparts.

Drawing on the 2014 panel of the Survey of Income and Program Participation (SIPP) data, in this study I investigated the self-rated health statuses and changes of individuals in same- and different-sex cohabitations and marriages over time (2014–2017). I focus on testing two competing hypotheses. Drawing on the minority stress theory and stress proliferation theory, I hypothesize that individuals in same-sex marriages and cohabitations will exhibit faster health decline compared to their counterparts in different-sex relationships. Conversely, informed by the gender-as-relational framework, I anticipate that individuals in same-sex marriages and cohabitations would have similar or even better health trajectories as compared to their different-sex peers [27–31].

## Method

### Data

This study used four waves of the 2014 Survey of Income and Program Participation (SIPP) data, which provide a recent, nationally representative sample of non-institutionalized civilian population in the United States. The U.S. Census Bureau administered the SIPP panel study to collect information on income, government program participation, and family dynamics. Wave 1 data of the SIPP 2014 panel were collected between February and June in 2014 from 72,160 individuals representing 29,685 households. Follow-ups with respondents were then scheduled annually from 2015 through 2017. Thus, the data track a large sample of individuals in same-sex and different-sex cohabitations and marriages, comprising a broad age range (17–88 years old), over four years (2014–2017). Time-varying information on respondents’ socioeconomic characteristics are also available in the data. The advantages of the 2014 SIPP include a direct measure of same-sex cohabiting and marital relationships, which reduces the chance of misclassification of individuals in same-sex relationships in prior surveys [32]; a relatively large sample of same-sex couples, which enables meaningful statistical comparisons between same- and different-sex couples; and annual data collection of self-rated health for four years, which allows longitudinal health trajectory comparisons.

### Sample

The present study focused on individuals in different-sex or same-sex married or cohabiting relationships at the beginning of the 2014 SIPP. Using the respondent’s household roster from the first month of the survey, I identified whether individuals were in different-sex or same-sex married or cohabiting relationships. Using respondent and partner identification numbers, I tracked whether couples continuously co-resided. Given the disruptive effects of union transition on health [33], I restricted the sample to individuals who stayed together with their partners over the 4-year study period to exclude dissolved relationships. This sample restriction also better suits the study goal to test gender-as-relational framework. Additional analyses including dissolved relationships (not shown but available upon request) suggest main results do not change due to this sample restriction. Because the Census Bureau allocates values through imputation procedures when data are missing, the amount of missing data in this study is small. I dropped observations containing missing values on any of the variables analyzed, which accounted for 0.02% of the total person-year observations. I further restricted the sample to individuals who completed at least two surveys.

The final sample included 20,804 individuals in different-sex marriages, 2,271 individuals in different-sex cohabiting relationships, 69 individuals in same-sex marriages, and 153 individuals in same-sex cohabiting relationships. The final sample size was 75,656 person-year observations. The sample attrition rate between waves 1 and 4 was 37%, which is comparable to previous SIPP panel attrition rates ranging from 31 to 43% [34]. Supplemental analyses (not shown but available upon request) using data from only individuals who completed all four waves produced substantively similar results.

### Measures

#### Dependent variable

The dependent variable is self-rated health (SRH). SRH was measured in the SIPP annually for four years from 2014 to 2017. A large body of literature suggests that SRH is a summary measure of overall health status and a strong predictor of morbidity and mortality [35, 36]. Respondents were asked to rate their general health as excellent, very good, good, fair, or poor. A higher score indicated better SRH. Prior research recommends using all five categories of SRH responses in longitudinal analyses as it enables a better capture of moderate changes over a short time period [34]. Therefore, I treated SRH as a continuous variable. As a robustness check, I modeled SRH as an ordinal variable or a dichotomous variable (1 = excellent/very good health; 0 = poor/fair/good health). The results revealed consistent patterns (not shown but available upon request).

#### Key independent variable

The key independent variable is relationship status. I constructed a variable indicating whether the respondent was in a different-sex married, different-sex cohabiting, same-sex married, or same-sex cohabiting relationship in the first month of the panel. Beginning with the 2014 SIPP, the Census Bureau implemented a new household roster that directly identifies same-sex cohabiting and married relationships in all households [37]. The household roster incorporated relationship options for “opposite-sex” or “same-sex” spouses and unmarried partners. This strategy represented a major gain over the traditional method as it measured same-sex relationships directly rather than relying on the sex composition of both partners to identify same-sex relationships. Thus, this measurement approach reduced the possibility of misclassification of same-sex relationships [32].

#### Control variables

I included several sociodemographic covariates to account for the potential confounders in the relationship between SRH and relationship status [5, 7]. The time-varying socioeconomic indicators included poverty status, health insurance coverage, and educational attainment. The measure of poverty was based on federal poverty level guidelines for each household size. Individual who reported an annual household income below 100% of the federal poverty line for a given household size was considered to be “in poverty”. Health insurance coverage was defined by whether or not the respondent was covered by any type of public or private health insurance (1 = Yes). Education included four categories: less than high school, high school, some college education, and college graduate, with less than high school as the reference group. The time in-variant demographic control variables included baseline age in years, sex, race-ethnicity (non-Hispanic White, non- Hispanic Black, Hispanic, Asian, and Other), nativity (1 = U.S. born), region of residence (Northeast, Midwest, South, and West), metropolitan status (1 = metro area), and the presence of children in the household (1 = household with at least one minor).

#### Analytic strategy

To examine initial health statuses and health changes of same- and different-sex, married and cohabiting people, I followed strategies used by prior research in analyzing health trajectories using SIPP [34]. I first estimated a mixed-effects linear regression to assess the SRH trajectories. To analyze whether the SRH changes of same- and different-sex, married and cohabiting individuals differed, I included interaction terms between time and a set of dummy variables indicating relationship type. To reduce selection bias from unmeasured time-invariant confounding factors, such as survey disclosure selection, relationship length prior to the study, and individual perception of health (i.e., individual inclination to view themselves as a healthy or unhealthy person), I further estimated a fixed-effects linear regression.

## Results

Table 1 provides the descriptive statistics of same-sex and different-sex, married and cohabiting individuals based on the first year of the panel data. Same-sex married people are younger, more likely to be highly educated, U.S. born, identify as White, have health insurance, and are less likely to be in poverty or have minor children in households than different-sex married counterparts. Same-sex cohabitors and different-sex married people look very similar in terms of poverty status and insurance coverage. Both same-sex cohabiting and married individuals are more likely to live in metropolitan areas and in the Northeast compared to different-sex married adults. Among the four groups, same-sex married individuals were most likely to report excellent or very good health in the first wave, followed by same-sex cohabiting, different-sex cohabiting, and different-sex married individuals. It is important to note that same-sex marriage was not legalized at the federal level in the U.S. until 2015 (Obergefell v. Hodges, 2015). The same-sex married individuals in this sample may be highly selected on certain observed (e.g., education and income) and unobserved (e.g., relationship commitment) characteristics, given the fact that they were able to marry when same-sex marriage was only available in certain states.

**Table 1:** Descriptive Statistics by Relationship Type at Wave 1

	Different-Sex Couples		Same-Sex Couples	
	Married	Cohabiting	Married	Cohabiting
Age	52.19	38.59	44.77	45.52
Male	0.50	0.50	0.41	0.31
Race				
Non-Hispanic White	0.72	0.59	0.75	0.76
Non-Hispanic Black	0.08	0.12	0.04	0.06
Hispanic	0.13	0.22	0.15	0.10
Asian	0.05	0.02	0.06	0.04
Other	0.02	0.05	0.00	0.04
Education				
Less than HS	0.12	0.19	0.07	0.03
HS Grad	0.29	0.35	0.20	0.21
Some College	0.26	0.28	0.19	0.27
College Grad	0.33	0.18	0.54	0.49
Nativity (U.S born)	0.82	0.82	0.91	0.93
Region				
Northeast	0.13	0.12	0.20	0.15
Midwest	0.22	0.21	0.30	0.29
South	0.44	0.41	0.41	0.29
West	0.21	0.26	0.09	0.27
Metro	0.76	0.75	0.88	0.86
Poverty	0.08	0.17	0.03	0.08
Have Health Insurance	0.88	0.65	0.93	0.89
Minor Child in Household	0.42	0.52	0.29	0.17
Self-Rated Health				
Poor	0.05	0.04	0.00	0.03
Fair	0.12	0.11	0.12	0.14
Good	0.29	0.29	0.19	0.20
Very Good	0.32	0.31	0.39	0.37
Excellent	0.22	0.25	0.30	0.26
*N*	20,804	2,271	69	153

To further examine the association of relationship type and self-rated health, I turned to the regression results. Results from the mixed-effects linear regression are presented in Table 2. The model controlled for basic demographic and socioeconomic variables and showed that same-sex married individuals do not differ significantly from different-sex married people in initial SRH. This result is consistent with prior work, finding no significant differences in health when comparing individuals in same-sex marriages to those in different-sex married unions [6, 38, 39]. Although same-sex cohabitors reported slightly worse SRH than different-sex married adults, the difference was not statistically significant. This is partly attributed to the selection of couples staying together over the four years in this study. The same-sex cohabiting relationships in this sample may be more “marriage-like” due to the selection of stable and committed relationships, thus more similar to marriages in promoting health. Additional analyses (shown in Appendix) using samples that did not select for stable relationships (couples who stayed together for four years) showed that the initial health status of same-sex cohabiting individuals was significantly lower than that of different-sex married people. Compared to different-sex married individuals, different-sex cohabitors were less likely to report good health in the base year, and the difference is significant between the two groups (coef = -0.08, *p* < 0.001).

**Table 2:** Mixed-Effects Linear Model of Self-Rated Health

	*b*		*SE*
Union type (ref = Different-Sex Married)			
Different-Sex Cohabiting	-0.08	***	0.02
Same-Sex Married	0.04		0.11
Same-Sex Cohabiting	-0.10		0.08
Time	-0.03	***	0.00
Time # Different-Sex Cohabiting	0.01		0.01
Time # Same-Sex Married	-0.15	***	0.04
Time # Same-Sex Cohabiting	-0.05		0.03
Age	-0.02	***	0.00
Male	0.03	**	0.01
Race (ref = Non-Hispanic White)			
Non-Hispanic Black	-0.20	***	0.02
Hispanic	-0.06	**	0.02
Asian	-0.17	***	0.03
Other	-0.22	***	0.03
Nativity (ref = U.S. born)	-0.09	***	0.02
Metro	0.10	***	0.01
Region (ref = Northeast)			
Midwest	-0.07	***	0.02
South	-0.14	***	0.02
West	-0.03		0.02
Minor Child in Household	0.00		0.01
Education (ref = Less than High School)			
High School Graduate	0.23	***	0.02
Some College	0.35	***	0.02
College Graduate	0.69	***	0.02
Poverty (ref = Not in Poverty)	-0.15	***	0.01
Have Health Insurance	0.03	*	0.01
Observations	75,656		
Number of individuals	23,297		

Unstandardized coefficients (*b*) reported with standard errors (*SE*). *p < .05, **p < .01, ***p < .001

**Fig. 1:**
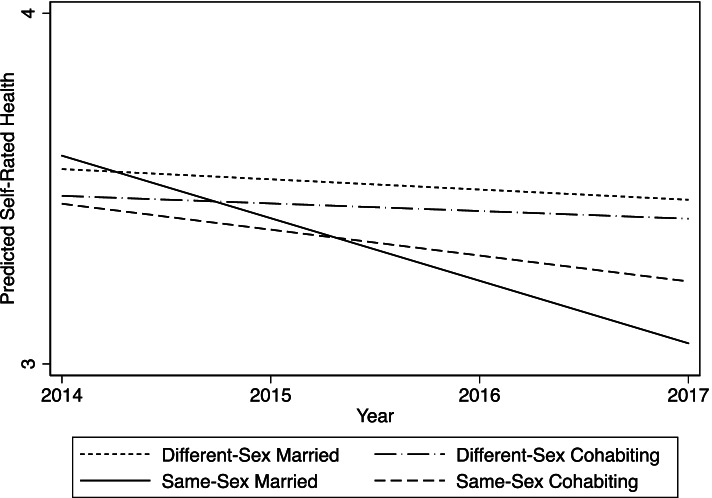
Estimated Health Trajectories Based on the Mixed-Effects Model in Table 2

Next, I turned to the health changes of same- and different-sex, cohabiting and married individuals from 2014–2017. All four groups showed declining health over the four-year period. The coefficient for the time variable represents the average annual SRH change rate for different-sex married people (coef = -0.03, *p* < 0.001), and the interaction between time and same-sex married status indicates the difference in the rate of change in health between same-sex married and different-sex married groups. The interaction between time and same-sex married status was negative and significant (coef = -0.15, *p* < 0.001). This finding suggests that although same-sex married individuals showed similar SRH compared to different-sex married counterparts in the base year, they reported faster health decline than different-sex married individuals. Additional results (not shown) from rotating the reference groups revealed that same-sex cohabitors also had faster health decline compared to different-sex cohabitors and the difference is marginally significant (*p* = 0.074). These patterns are summarized in Fig. 1, which demonstrates the steepest decline rate for same-sex married individuals, followed by same-sex cohabiting, different-sex married, and different-sex cohabiting people.

Table 3 shows results from the fixed-effects linear model. This model further adjusted for time-invariant unmeasured confounders by using each individual as their own control. Similar to results from the mixed-effects regressions, the fixed-effects model results tell a consistent story: while SRH declined for all four groups over time, the rate of decline was faster for same-sex married individuals (significant negative interaction term). The inclusion of both mixed-effects and fixed-effects models, along with a series of sensitivity tests and supplementary analyses, suggest that the results of this study are robust to sample selection, model specification, panel attrition, different categorizations of the outcome variable, and unobserved heterogeneity.

**Table 3:** Fixed-Effects Linear Model of Self-Rated Health

	*b*		*SE*
Time	-0.03	***	0
Time # Different-Sex Cohabiting	0.01		0.01
Time # Same-Sex Married	-0.16	***	0.04
Time # Same-Sex Cohabiting	-0.04		0.03
Education (ref = Less than High School)			
High School Graduate	0.03		0.03
Some College	-0.02		0.04
College Graduate	0.06		0.05
Minor Child in Household	-0.02		0.02
Metro	0		0.04
Region (ref = Northeast)			
Midwest	0.07		0.1
South	0.2	*	0.09
West	0.11		0.1
Poverty (ref = Not in Poverty)	-0.05	***	0.01
Have Health Insurance	0.04	**	0.01
Observations	75,656		
Number of individuals	23,297		

Unstandardized coefficients (*b*) reported with standard errors (*SE*). *p < .05, **p < .01, ***p < .001

## Discussion

This study assesses health changes over time among individuals in same- and different-sex married and cohabiting unions in the United States. Using nationally representative longitudinal data, I demonstrated that individuals in same-sex marital relationships experienced an elevated risk of health decline between 2014–2017 compared to those in different-sex marriages. This distinctive finding supports the minority stress proliferation framework, suggesting that minority stress may accumulate over time and “get under the skin” of same-sex couples, resulting in their more precipitous health deterioration. The current study adds to the expanding literature on minority status, intimate relationships, and health by providing empirical insights into our understanding of how being in a socially disadvantaged romantic relationship contributes to changes in health.

Despite the important prior work in documenting same-sex couples’ health using nationally representative data, critical gaps remain in understanding whether and to what extent same-sex relationship is associated with health changes over time at the population level. This study goes beyond previous work to systematically assess same-sex relationships and health changes during the period spanning from 2014 through 2017. SIPP’s longitudinal follow-up of same-sex couples represents a major advantage over previous cross-sectional analyses and provides the first opportunity to tap into health changes of individuals in same-sex relationships in nationally representative data. Same-sex cohabitors and married adults both exhibited a more rapid decline in self-rated health than their different-sex counterparts. This pattern is evident even after accounting for some selection bias in the fixed-effects model. The faster health decline among individuals in same-sex relationships observed in this study provides support for the minority stress and stress proliferation theories—being in an intimate relationship that has been long devalued and disrespected by society, and facing discrimination and stigma on a daily basis, is stressful. While these analyses were unable to directly test whether individual experience of discrimination and stigma to account for the health decline pattern (due to the lack of such measures in the SIPP), future studies should consider the unique stressors that same-sex couples experience to better understand their health changes.

It is important to note that the 2014 SIPP covers a period (2014–2017) during which significant social and legal changes concerning same-sex couples occurred. The U.S. Supreme Court ruling provided same-sex couples with the constitutional right to marry in 2015, thus legalizing same-sex marriage in all states. One may have expected that the legalization process would benefit same-sex couples’ health through greater access to institutional resources, reduced institutionalized discrimination, and increased public acknowledgment [40]. However, the pattern of more rapid health decline among individuals in same-sex relationships indicates that the beneficial effects of the legal changes, if any, may not be enough to offset the negative health effects of discrimination against same-sex couples [41]. It is also likely that the unfolding presidential campaign and ultimate election of Trump during the 2014–2017 period added another layer of stress to sexual minorities, especially same-sex married couples. Same-sex married people had potentially more to fear or lose given the Trump administration’s homophobic policies, including a potential rollback of the same-sex marriage ban, a reversal of antidiscrimination laws protecting sexual minorities’ employment, sexual, and reproductive rights, allowing businesses to discriminate against sexual minorities (e.g., Masterpiece Cakeshop Ltd. v. Colorado Civil Rights Commission), and excluding sexual orientation and gender identity questions from national surveys [42–44].

### Limitations

There are several limitations to note in this study. First, the study is limited to examining self-rated health changes over a span of four years. We should caution against generalizing the finding to a longer time frame that is outside of the study period (2014–2017). Since the sample sizes for same-sex cohabiting and married people are relatively small, misclassification errors may bias results. However, such misclassification usually bias results toward the null hypothesis. Second, I was unable to identify and disentangle the impact of different processes (e.g., individual experience of discrimination, societal and political changes) that contribute to the accelerated health decline of same-sex married and cohabiting individuals. I recommend future studies explore what mechanisms explain this health decline pattern. Third, it was not possible with the present data to distinguish between men and women in same-sex relationships. The small sample size and thus the lack of sufficient statistical power prevented the study from stratifying analysis by sex. Nevertheless, it is worth noting that lesbian women experience more health disadvantages relative to gay men due to the combination of gender and sexual minority statuses, according to existing research [38, 45]. A population-based longitudinal dataset with a larger sample of men and women in same-sex relationships is needed to consider possible gender variations. Finally, the 2014 SIPP does not include measures of sexual orientation and non-binary gender identity. Therefore, findings from the current study cannot be generalized to unpartnered sexual minorities, gender non-conforming and transgender people. Future research with more comprehensive measures of sexual orientation, gender identity, and relationship type should further explore whether coupling affects unpartnered sexual minorities, gender non-conforming and transgender individuals’ health changes differently than it does gay and lesbian people.

## Conclusions

The present study provides one of the first nationally representative longitudinal analysis of same-sex relationships and health changes over time. The current findings underscore the importance of considering the health of individuals in marginalized intimate relationships in relation to the changing social and legal contexts from a dynamic point of view. The complexity of health changes of same-sex married and cohabiting people calls for policymakers’ attention to focus on the social processes that contribute to the health of sexual minority populations. Although the U.S. has seen significant social and legal changes regarding marriage equality in recent decades, critical gaps remain in our understanding of how structural-level policy changes and couple-level relationship dynamics shape the health of same-sex partners over time. Given the importance of heterosexual marriage for health, recent institutionalization of same-sex marriage in the U.S., and the minority stress and health disparities experienced by gays and lesbians, it is imperative that we use nationally representative longitudinal data to advance scientific knowledge about the relationship experiences of same-sex partners in relation to change in their health over time. While this study demonstrates the value of longitudinal data in studying sexual minorities’ health, future longitudinal research and data collection efforts are necessary to achieve a deeper and more complete understanding of same-sex relationships and health as the social and legal contexts continue to change.

## Data Availability

The datasets used and analyzed during the current study are available at the census website (https://www.census.gov/programs-surveys/sipp/data/datasets/2014-panel.html).

## Appendix

Table 4

**Table 4:** Mixed-Effects and Fixed-Effects Linear Models of Self-Rated Health (including all individuals in relationships in wave 1)

	Mixed-Effects Model			Fixed-effects Model		
	*b*		*SE*	*b*		*SE*
Union type (ref = Different-sex married)						
Different-Sex Cohabiting	-0.09	***	0.02			
Same-Sex Married	0.03		0.11			
Same-Sex Cohabiting	-0.17	*	0.07			
Time	-0.03	***	0	-0.03	***	0
Time # Different-Sex Cohabiting	0		0.01	0		0.01
Time # Same-Sex Married	-0.16	***	0.04	-0.17	***	0.04
Time # Same-Sex Cohabiting	-0.02		0.02	-0.01		0.02
Age	-0.02	***	0			
Male	0.03	**	0.01			
Race (ref = Non-Hispanic White)						
Non-Hispanic Black	-0.2	***	0.02			
Hispanic	-0.06	**	0.02			
Asian	-0.17	***	0.03			
Other	-0.22	***	0.03			
Nativity (ref = U.S. born)	-0.1	***	0.02			
Metro	0.09	***	0.01	-0.02		0.04
Region (ref = Northeast)						
Midwest	-0.07	***	0.02	0.08		0.1
South	-0.14	***	0.02	0.24	**	0.08
West	-0.03		0.02	0.11		0.1
Education (ref = Less than High School)						
High School Graduate	0.23	***	0.02	0.03		0.03
Some College	0.34	***	0.02	-0.05		0.04
College Graduate	0.7	***	0.02	0.04		0.05
Minor Child in Household	0		0.01	-0.02		0.02
Poverty (ref = not in poverty)	-0.15	***	0.01	-0.05	***	0.01
Have Health Insurance	0.02		0.01	0.03		0.01
Union transition (ref = no transition)						
Cohabitation to married	0.08	**	0.02	0.06	*	0.03
Cohabitation to single	-0.04		0.03	-0.01		0.04
Single to cohabitation	-0.05		0.07	-0.03		0.08
Single to married	-0.23	**	0.07	-0.21	**	0.08
Married to single	0.01		0.03	0.04		0.03
Married to cohabitation	0		0.03	0		0.03
Observations	81,097			81,097		
Number of individuals	25,132			25,132		

Unstandardized coefficients (b) reported with standard errors (SE). **p* < .05, ***p* < .01, ****p* < .001

## Abbreviations

SIPP: Survey of Income and Program Participation
SRH: Self-Rated Health
